# 

**Published:** 1790

**Authors:** 


### CONTENTS.

Page

I. *A Case of Diabetes. By Mr.* Philip Werner, *Surgeon of the British Factory at Algiers.* — — — 221

II. *Description of an improved Instrument for the Fistula in Ano. By Mr.* J. Savigny, *Surgical-Instrument Maker in London.* 228

III. *An Account of two Cases of Amenorrhœa, with some Observations on the Use of the Root of Madder in that Disease. By Mr.* Peter Copland, *Surgeon at Swayfield, near Colsterworth, in Lincolnshire.* — — 230

IV. *An Account of two Cases of Pemphigus; to which is added a Fact relative to the early Practice of Inoculation of the Small Pox in Wales. By Mr.* John Ring, *Surgeon in London.* — — — — — 234

V. *Observations on Stone in the urinary Bladder, and on Lithotomy. By Mr.* James Lucas, *one of the Surgeons of the General Infirmary at Leeds.* — — — 237

VI. *Farther Remarks on the Treatment of Phthisis Pulmonalis. By* William May, *M. D. Extra-Licentiate of the College of Physicians, London; and Physician at Maidstone in Kent.* — — — — 255

Page

VII. *Observations on the Luxation of the Bones of the Pelvis. By M.* Enaux, *Professor of Midwifery, one of the Surgeons of the General Hospital, and Member of the Academy of Sciences at Dijon.*—Vide *Nouveaux Memoires de l'Academie de Dijon, pour la Partie des Sciences et Arts,* Vol. III. 265

VIII. *Observations on the Diseases and medical Practice of Boutan and Thibet; being Part of a Paper entitled "Some Account of "the vegetable and mineral Productions of "Boutan and Thibet; by Mr.* Robert Saun- "ders, *Surgeon at Boglepoor in Bengal;" inserted in the Philosophical Transactions of the Royal Society of London,* Vol. LXXIX. *for the Year* 1789, Part I. — — 274

IX. *A physical Inquiry into the Powers and Operation of Medicines. By* Thomas Percival, *M. D. F. R. S. and S. A. Lond. F. R. S. and R. M. S. Edinb. &c.*—*From the Memoirs of the Literary and Philosophical Society at Manchester, Volume the Third.* 287

Catalogue *of* Books. — — 308

